# Role of Ultrasonography in the Evaluation of Normal Developmental Pattern of Fetal Cerebral Sulci Between 18 and 32 Weeks of Gestational Age

**DOI:** 10.7759/cureus.22581

**Published:** 2022-02-24

**Authors:** Chaithanya A, Anil K Sakalecha, Srinivasa B.C.R.

**Affiliations:** 1 Radiodiagnosis, Sri Devaraj Urs Academy of Higher Education and Research, Kolar, IND; 2 Radiodiagnosis, Sapthagiri Institute of Medical Sciences and Research Centre, Bangalore, IND

**Keywords:** ultrasonography, gestation, fetal cerebral sulci, brain development, antenatal

## Abstract

Background

Brain development is a crucial process of intrauterine life and can be readily visualized on ultrasonography. This study aims to visualize developmental patterns of various fetal cerebral sulci using ultrasonography between 18 and 32 weeks of gestation. Sulci are best visualized on images that are taken perpendicular to their expected course of development. Initially, they appear as small dot/dimple on the brain surface and later develop into a V-shaped indentation and finally deepen to form notch and echogenic line into the brain forming a Y-shaped configuration.

Material and methods

This was a cross-sectional observational study conducted on 241 antenatal mothers with a singleton pregnancy between 18 and 32 weeks of gestational age. Demographic and clinical data were obtained. Ultrasonography was performed using PHILIPS EPIQ 5, a curvilinear probe of frequency 2-5MHz. Sulci/fissures that are reported to appear early on anatomical studies were evaluated, specifically the parieto-occipital fissure, calcarine fissure, cingulate sulci, insula/Sylvian fissure, and convexity sulci. Comparison of the categorical outcomes was performed between study groups using the chi-square test. A p-value of <0.05 was considered statistically significant.

Results

The study included 241 participants. The mean age of antenatal mothers was 24.09 ± 4.13 years, and the mean fetal gestational age was 24.99 ± 4.13 weeks. Parieto-occipital fissure was the first fissure to develop and was present as a V-shaped indentation at 18 weeks and as a Y-shaped configuration by 21-22 weeks. Calcarine fissure was the next fissure to appear; it appeared as a dot by 18 weeks and developed into a V-shaped indentation by 20 weeks and as a Y-shaped configuration by 23 weeks. All fissures except cingulate sulci had appeared by 20 weeks, and calcarine sulci appeared later in the gestation by 21 weeks. Sylvian fissure initially appeared as a smooth surface and later underwent operculization to form obtuse and acute angulations with the adjacent temporal lobe by 20 and 24 weeks, respectively. Convexity sulci appeared later in gestation, beyond 25-26 weeks.

Conclusions

Ultrasonography, which is the commonest modality used in antenatal assessment of the fetus, can also be used to identify, familiarize, and provide a standard reference to assess normality of fetal sulcations. Neuronal migration disorders result in a wide spectrum of malformations of cortical development whose clinical manifestations include severe psychomotor retardation, developmental delays, motor deficits, seizures, and failure to thrive. Knowledge of normal development patterns of fetal cerebral sulci helps in early suspicion and detection of these cortical malformations, when present.

## Introduction

The human brain is the seat of highly evolved intelligence that sets us apart from other species. Human nervous system is one of the most complex, widely investigated, and yet poorly understood physical system known to mankind. Structural appearance of the human brain and its functions are inseparable from daily activities of life like physical, mental, intellectual, and cultural aspects. The brain is accommodated in a limited space, the cranial box. The cerebral cortex within is folded into numerous convolutions called the gyri and separated by multiple depressions called the sulci. The total surface area of the brain cortex is estimated to be around 2,200 cm2, of which only one-third is visible on the surface and rest is obscured [1]. Brain development in a fetus is a complicated yet a highly organized and progressive process that continues throughout pregnancy, with intermittent periods of rapid brain growth [2].

The knowledge of sulci and gyri is not only helpful in differentiating the sensory and motor areas of the brain (e.g.: central sulcus is a limiting sulcus) but also very crucial in diagnosing the malformations of cortical development. These sulci appear in an orderly sequence, but they vary in dimensions in different individuals and also when compared to the left and right hemispheres of the same individual [1]. By the end of pregnancy, the fetal brain is a complex array of sulci (furrows) and gyri (ridges) looking much like an adult brain.

Neuronal migration disorders result when the post-mitotic neurons fail to migrate to the cortical plate, resulting in a wide spectrum of cortical malformations whose clinical manifestations include severe psychomotor retardation, developmental delay, seizures, and failure to thrive. The term “malformations of cortical development” was designed to address these collective groups of brain developmental disorders. For example, lissencephaly (smooth brain) is a neurological disorder characterized by only a few shallow sulci on the cerebral surface and has a deleterious prognosis. Early diagnosis of these conditions is crucial for appropriate antenatal counselling and optimization of obstetric management [3,4]. Assessment of fetal sulcal development to understand the cortical maturation through sonography has now become widespread. Traditionally, transabdominal two-dimensional (2D) ultrasonography is used as the main method to assess fetal cerebral sulcation [4]. More recently, three-dimensional (3D) ultrasonography and magnetic resonance imaging (MRI) have also emerged as assessment tools for fetal cerebral fissures [5-7]. However, 2D ultrasonography still remains the most common, convenient, and effective method for prenatal examination.

Review of literature

A prospective cross-sectional study conducted by Pingping et al. [8] on 845 normal fetuses between 21 and 32 weeks of gestation studied the depth and width of the Sylvian fissure (SF) in relation to the gestational age and concluded that the depth of SF positively correlated with gestational weeks, whereas the width of SF was negatively correlated with gestational weeks, with correlation coefficients 0.751 and 0.825, respectively (all p < 0.001).

Another study by Toi et al. [9] studied the earliest gestation at which fetal cerebral sulci were visible on prenatal ultrasonography and reported that major cerebral sulci were visualized as early as 18 weeks’ gestation. Parieto-occipital fissure (POF) and calcarine sulcus were seen earliest at 18 weeks, and cingulate and convexity sulci were seen at 23 weeks’ gestation. Gestational ages at which particular sulci were always visible were as follows: POF at >20 weeks, calcarine sulcus at >21 weeks, cingulate sulcus (CS) at >24 weeks, and convexity sulci at >27 weeks. These data were also consistent with previous anatomical and fetal MRI studies.

Currently, only limited information is available regarding early developmental stages of focal anomalies of fetal cortical gyration. Despite the potential value of such an assessment, only a scarce number of studies with small numbers of individuals have described normal gyration. There has been enormous data on fetal biometric parameters such as biparietal diameter (BPD), head circumference (HC), abdominal circumference (AC), and femur length (FL) to predict accurate gestational age. Furthermore, correlation of this fetal biometrics with developmental patterns of fetal cerebral gyrations can be made with utmost precision. Globally, studies are exploring the use of both ultrasonography and MRI in correlating the cortical and gyral developments with the gestational age. However, studies conducted in India are still obscure. Perhaps, due to a small sample size and lack of standardization of ultrasonographic views, these studies showed significant variation in fetal sulcus development [7,10,11]. Therefore, this study aimed to evaluate the patterns of various fetal cerebral sulci using ultrasonography between 18 and 32 weeks of gestational age to depict the chronological order of their appearance and to correlate the appearance with their gestational age.

This article was previously presented as a meeting abstract at the 2021 Sonosummit Virtual Conference organized by the Indian Radiological and Imaging Association on August 29, 2021.

## Materials and methods

Source of data

This study was a cross-sectional observational study conducted on 241 antenatal mothers with a singleton pregnancy between 18 and 32 weeks of gestational age who underwent routine antenatal ultrasonography at the Department of Radiodiagnosis, R. L. Jalappa Hospital and Research Center, attached to Sri Devaraj Urs Medical College, Kolar. The study was approved by the Institutional Ethics Committee, Sri Devaraj Urs Medical College, Kolar (No. SDUMC/KLR/IEC/133/2019-20). Informed written consent was attained from all the study participants, and only those participants willing to sign the informed consent were included in the study. The benefits and risks involved in the study and voluntary nature of participation were explained to the participants before obtaining consent. Confidentiality of the study participants was maintained.

Singleton pregnancies with dating of pregnancy based on sonography performed before the 13th week of gestation were included in the study. Exclusion criteria included mothers with a history of smoking, alcohol intake, drug abuse, intake of any category X drugs, and previous pregnancy with fetal congenital anomalies or fetal central nervous system (CNS) malformations.

Methods of data collection

All the statutory requirements under Pre-Conception and Pre-Natal Diagnostic Techniques Act were followed and form F duly completed. Informed written consent was obtained from all the study participants for their willingness to participate in the study. Patients fulfilling the inclusion criteria were taken as the study population, and their details, clinical history, physical examination, and investigations were recorded in a structured pre-prepared performa. Details of the study protocol were explained to the patients. Gestational age was established by at least one of the antenatal ultrasonography scans performed before 13 weeks of gestation.

The study was then performed using a high-end ultrasound machine, Philips EPIQ 5 using a curvilinear probe of frequency 2-5 MHz. Present gestational age was determined by fetal ultrasound biometry (average of BPD, HC, AC, and FL) and was correlated with gestational age as per dating scan or scans performed before 13 weeks of gestation. Those sulci and fissures that were reported to appear relatively early on anatomical studies were evaluated, specifically Parieto-occipital fissure (POF), Calcarine fissure (CF), Cingulate sulcus (CS), Convexity sulci, and insula/ Sylvian fissure (SF) [4]. A standard axial view of the fetal head was obtained at the level of BPD, and then a slight cephalad adjustment was made to obtain a plane closer to the upper margin of the occipital horns of lateral ventricles, where the POF was visualized in the transventricular plane. Then the ultrasound probe was turned 90° to obtain a coronal view of posterior fossa where the CF was imaged in a transcerebellar plane. CS was best imaged in a coronal plane above the region of thalami, where cavum septum pellucidum (CSP) was seen. Once imaged, these fissures/sulci were graded as dot, V-shaped pattern, or Y-shaped pattern based on their appearance. SF was assessed in the axial plane by making a small caudal adjustment to the standard plane of BPD. Then the insular margin was decided to be either smooth or angular. Whenever it was angular, the angle was again defined to be obtuse if the angle between the adjacent temporal lobe operculum and the insula was greater than 90° and acute if the angle was less than 90°. Convexity sulci were visualized in the axial plane and were said to be present if indentations were noted on the lateral brain surface [9]. Once the various patterns of appearance of these fetal cerebral sulci were recorded, they were correlated with the gestational age of the fetus. This was a single observer study, and it was noted that the sulci were best visualized in image planes obtained perpendicular to the expected course of a particular sulcus and in a favorable fetal position; other than that, no notable intraobserver difference was found.

At a given gestational age, a particular sulcus was considered to be present when it was observed in more than 75% of the fetuses, detectable if it was observed in 25-75% of the examinations, and absent when it was observed in less than 25% of the examinations [12].

POF, CF, CS, SF, and convexity sulci were considered as primary outcome variables. Period of gestation as per ultrasonography was considered as the primary explanatory variable. Descriptive analysis was carried out using mean and standard deviation for quantitative variables and using frequency and proportion for categorical variables. Categorical outcomes were compared between study groups using the chi-square test. A p-value of <0.05 was considered statistically significant. Data were analyzed using coGuide software, V.1.03.71.

## Results

A total of 241 participants were included in the final analysis of our study. Among them, 84 (34.85%) antenatal mothers were between 18 and 22 years of age, 102 (42.32%) between 23 and 27 years of age, 42 (17.43%) between 28 and 32 years of age, and 13 (5.39%) between 33 and 37 years of age. Majority of the antenatal mothers belonged to the age group of 23 to 27 years (n=102; 42.32%) (Table 1). Among them, 112 (46.47%) were primigravida and 129 (53.53%) were multigravida (Table 1), and 26 (10.79%) had a previous history of abortions.

**Table 1:** Summary of baseline parameter of study participants (N=241) USG, ultrasonography

Parameter	Summary
Maternal age (in years)	24.09 ± 4.13 (range: 18 to 37)
Maternal age group	
18–22 years	84 (34.85%)
23–27 years	102 (42.32%)
28–32 years	42 (17.43%)
33–37 years	13 (5.39%)
Gravida status of antenatal mothers	
Primigravida	112 (46.47%)
Multigravida	129 (53.53%)
Periods of gestation	
Gestational age as per dating scan (in weeks)	24.99 ± 4.21 (range: 17.57 to 33.71)
Gestational age by USG (in weeks)	24.99 ± 4.13 (range: 18 to 32)
Gestational age	
18 weeks–19 weeks 6 days	35 (14.52%)
20 weeks–21 weeks 6 days	35 (14.52%)
22 weeks–23 weeks 6 days	35 (14.52%)
24 weeks–25 weeks 6 days	31 (12.86%)
26 weeks–27 weeks 6 days	35 (14.52%)
28 weeks–29 weeks 6 days	35 (14.52%)
30 weeks–32 weeks	35 (14.52%)

Among 241 fetuses, 35 (14.52%) fetuses were studied between 18 weeks and 19 weeks 6 days, 20 weeks and 21 weeks 6 days, 22 weeks and 23 weeks 6 days, 26 weeks and 27 weeks 6 days, 28 weeks and 29 weeks 6 days, and 30 and 32 weeks gestation each, and 31(12.86%) fetuses were studied between 24 weeks and 25 weeks 6 days. The mean period of gestation as per dating scan was 24.99 ± 4.21 weeks (range: 17.57 to 33.71 weeks), and as per the present ultrasound it was 24.99 ± 4.13 weeks (range: 18 to 32 weeks) (Table 1).

Parieto-occipital fissure

**Figure 1:**
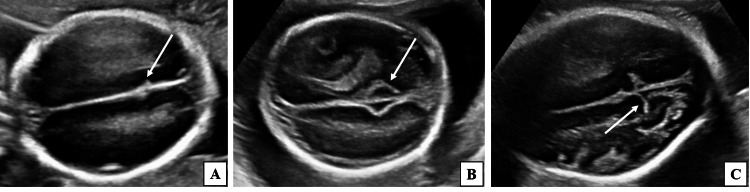
Two-dimensional ultrasound grey-scale images (axial sections) of the fetal head demonstrating various patterns of POF at different gestational ages. (A) Fetus of 18 weeks 1 day gestational age demonstrating POF in the form of a dot pattern (long white arrow). (B) Fetus of 20 weeks 2 days gestational age demonstrating POF in the form of V-shaped indentation (long white arrow). (C) Fetus of 26 weeks 5 days gestational age demonstrating POF in the form of Y-shaped configuration (long white arrow). POF, parieto-occipital fissure

Among the study fetuses, POF appeared as a dot in one (0.41%) fetus, as a V-shaped pattern in 65 (26.97%) fetuses, and as a Y-shaped pattern in the rest 175 (72.61%) fetuses. POF was always present between 18 weeks and 19 weeks 6 days of gestation as a V-shaped fissure, later was detectable as a Y-shaped fissure by 21-22 weeks, and was always present as a Y-shaped fissure after 24 weeks (Table 2 and Figure 1).

**Table 2:** Descriptive analysis of parieto-occipital fissure appearance and its correlation with gestational age in the study population (N=241) W, weeks; D, days

Period of gestation (in weeks and days)	Parieto-occipital fissure		
	Dot pattern (n=1; 0.41%)	V-shaped pattern (n=65; 26.97%)	Y-shaped pattern (n=175; 72.61%)
18W-19W+6D (N=35)	1 (2.86%)	32 (91.43%)	2 (5.71%)
20W-21W+6D (N=35)	-	18 (51.43%)	17 (48.57%)
22W-23W+6D (N=35)	-	15 (42.86%)	20 (57.14%)
24W-25W+6D (N=31)	-	-	31 (100%)
26W-27W+6D (N=35)	-	-	35 (100%)
28W-29W+6D (N=35)	-	-	35 (100%)
30W-32W (N=35)	-	-	35 (100%)

Calcarine fissure

**Figure 2:**
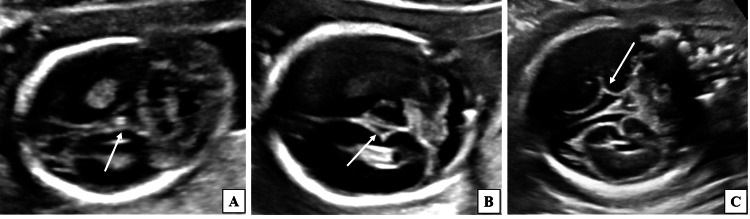
Two-dimensional ultrasound grey-scale images (coronal sections) of the fetal head demonstrating various patterns of CF at different gestational ages. (A) Fetus of 19 weeks 3 days gestational age demonstrating CF in the form of a dot pattern (long white arrow). (B) Fetus of 21 weeks 2 days gestational age demonstrating CF in the form of a V-shaped indentation (long white arrow). (C) Fetus of 25 weeks 6 days gestational age demonstrating CF in the form of a Y-shaped configuration (long white arrow). CF, calcarine fissure

CF appeared as a dot pattern in 28 (11.62%) fetuses, as a V-shaped pattern in 71 (29.46%) fetuses, and as a Y-shaped pattern in the rest 142 (58.92%) fetuses. CF was also present by 18 weeks to 19 weeks 6 days as a dot pattern and later progressed to appear as a V-shaped indentation by 20 weeks to 21 weeks 6 days and was detectable as a Y-shaped configuration by 22 weeks to 23 weeks 6 days (Table 3 and Figure 2).

**Table 3:** Descriptive analysis of calcarine fissure appearance and its comparison with gestational age in the study population (N=241). W, weeks, D, days

Period of gestation (in weeks and days)	Calcarine fissure		
	Dot pattern (n=28; 11.62%)	V-shaped pattern (n=71; 29.46%)	Y-shaped pattern (n=142; 58.92%)
18W-19W+6D (N=35)	28 (80%)	7 (20%)	-
20W-21W+6D (N=35)	-	33 (94.29%)	2 (5.71%)
22W-23W+6D (N=35)	-	24 (68.57%)	11 (31.43%)
24W-25W+6D (N=31)	-	7 (22.58%)	24 (77.42%)
26W-27W+6D (N=35)	-	-	35 (100%)
28W-29W+6D (N=35)	-	-	35 (100%)
30W-32W (N=35)	-	-	35 (100%)

Cingulate sulci

**Figure 3:**
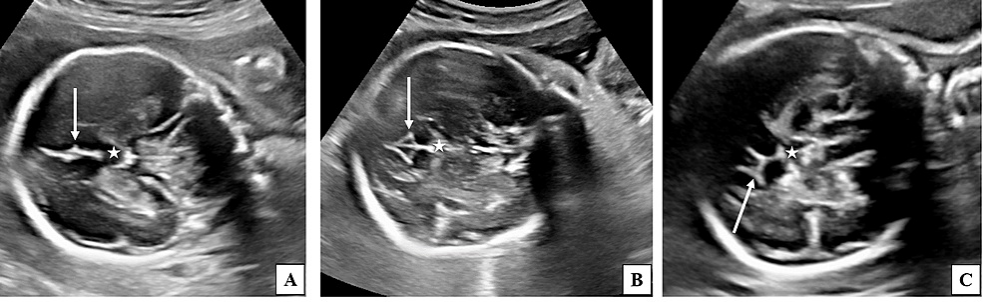
Two-dimensional ultrasound grey-scale images (coronal sections) where cavum septum pellucidum (star) was seen, demonstrating various patterns of CS at different gestational ages. (A) Fetus of 23 weeks 1 day gestational age demonstrating CS in the form of a dot pattern (long white arrow). (B) Fetus of 25 weeks 3 days gestational age demonstrating CS in the form of a V-shaped indentation (long white arrow). (C) Fetus of 28 weeks gestational age demonstrating CS in the form of a Y-shaped configuration (long white arrow). CS, cingulate sulcus

CS had not yet appeared in 57 (23.65%) fetuses, appeared as a dot pattern in 72 (29.88%) fetuses, as a V-shaped pattern in 53 (21.99%) fetuses, and as a Y-shaped pattern in the rest 59 (24.48%) fetuses. CS was seen later in the gestation by 20 weeks to 21 weeks 6 days as a dot, then formed a V-shaped indentation by 24 weeks to 25 weeks 6 days and finally developed into a Y-shaped configuration by 28 weeks to 29 weeks 6 days (Table 4 and Figure 3).

**Table 4:** Descriptive analysis of cingulate sulci appearance and its comparison with gestational age in the study population (N=241). W, weeks; D, days

Period of gestation (in weeks and days)	Cingulate sulci			
	Not yet appeared (n=57; 23.65%)	Dot pattern (n=72; 29.88%)	V-shaped pattern (n=53; 21.99%)	Y-shaped pattern (n=59; 24.48%)
18W-19W+6D (N=35)	29 (82.86%)	6 (17.14%)	-	-
20W-21W+6D (N=35)	23 (65.71%)	12 (34.29%)	-	-
22W-23W+6D (N=35)	5 (14.29%)	25 (71.43%)	5 (14.29%)	-
24W-25W+6D (N=31)	-	13 (41.94%)	18 (58.06%)	-
26W-27W+6D (N=35)	-	14 (40%)	16 (45.71%)	5 (14.29%)
28W-29W+6D (N=35)	-	2 (5.71%)	14 (40%)	19 (54.29%)
30W-32W (N=35)	-	-	-	35 (100%)

Sylvian fissure

**Figure 4:**
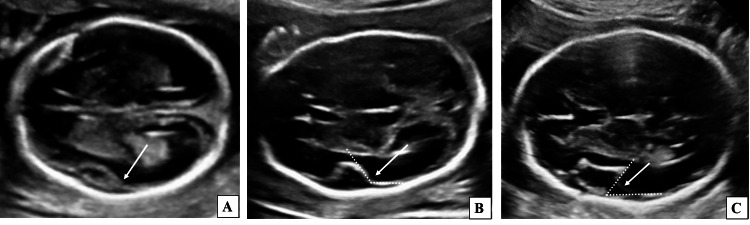
Two-dimensional ultrasound grey-scale images (axial sections) of the fetal head demonstrating operculization patterns of SF at different gestational ages. (A) Fetus of 19 weeks 4 days gestational age demonstrating SF in the form of a smooth pattern (long white arrow). (B) Fetus of 22 weeks gestational age demonstrating an obtuse angle (> 90°) between SF and the adjacent temporal lobe (long white arrow). (C) Fetus of 25 weeks 6 days gestational age demonstrating an acute angle (< 90°) between Sylvian fissure and the adjacent temporal lobe (long white arrow). SF, Sylvian fissure

SF appeared as a smooth surface in 42 (17.43%) fetuses, as an obtuse angle with the adjacent temporal lobe in 91 (37.76%) fetuses, and as an acute angle with the adjacent temporal lobe in the remaining 108 (44.81%) fetuses. SF appeared as a smooth surface at 18 weeks to 19 weeks 6 days; it then operculized to form an obtuse angle with adjacent temporal lobe by 20 weeks to 21 weeks 6 days and then operculized further to start forming an acute angle with the adjacent temporal lobe by 24 weeks to 25 weeks 6 days (Table 5 and Figure 4).

**Table 5:** Descriptive analysis of Sylvian fissure appearance and its comparison with gestational age in the study population (N=241). W, weeks; D, days

Period of gestation (in weeks and days)	Sylvian fissure		
	Smooth surface (n=42; 17.43%)	Obtuse angle (n=91; 37.76%)	Acute angle (n=108; 44.81)
18W-19W+6D (N=35)	26 (74.29%)	9 (25.71%)	-
20W-21W+6D (N=35)	16 (45.71%)	19 (54.29%)	-
22W-23W+6D (N=35)	-	33 (94.29%)	2 (5.71%)
24W-25W+6D (N=31)	-	19 (61.29%)	12 (38.71%)
26W-27W+6D (N=35)	-	11 (31.43%)	24 (68.57%)
28W-29W+6D (N=35)	-	-	35 (100%)
30W-32W (N=35)	-	-	35 (100%)

Convexity sulci

**Figure 5:**
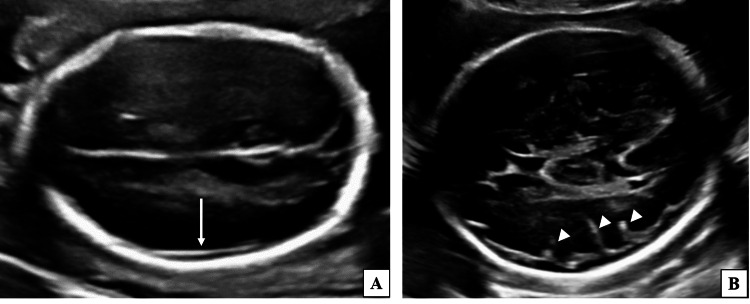
Two-dimensional ultrasound grey-scale images (axial sections) of the fetal head demonstrating the appearance of convexity sulci. (A) Fetus of 21 weeks 3 days gestational age showing a smooth lateral brain surface without any sulcal indentations (long white arrow). (B) Fetus of 28 weeks 5 days gestational age demonstrating multiple indentations on the lateral brain surface (white arrowheads) suggestive of convexity sulci.

Out of 241 participants, 99 (41.08%) had developed convexity sulci. Convexity sulci were visualized later in the gestation, beyond 26 weeks to 27 weeks 6 days. Details of the appearance of convexity sulci with increasing gestational age are described in Table 6 and Figure 5.

**Table 6:** Descriptive analysis of convexity sulci and its comparison with gestational age in the study population (N=241). W, weeks; D, days

Period of gestation (in weeks and days)	Convexity sulci	
	Not yet appeared (n=142; 58.92%)	Present (n=99; 41.08%)
18W-19W+6D (N=35)	35 (100%)	-
20W-21W+6D (N=35)	35 (100%)	-
22W-23W+6D (N=35)	35 (100%)	-
24W-25W+6D (N=31)	24 (77.42%)	7 (22.58%)
26W-27W+6D (N=35)	13 (37.14%)	22 (62.86%)
28W-29W+6D (N=35)	-	35 (100%)
30W-32W (N=35)	-	35 (100%)

Minimum gestational age of visualization of POF and CF was 18 weeks to 19 weeks 6 days, for CS it was 20 weeks to 21 weeks 6 days, for SF it was 20 weeks to 21 weeks 6 days, and for convexity sulci it was 25 weeks and beyond (Table 7).

**Table 7:** Comparison of minimum POG (in weeks and days) of appearance of various sulci (N=241). POG, period of gestation

Sulci	Minimum POG of visualization
Parieto-occipital fissure	18 weeks to 19 weeks 6 days (V-shaped pattern)
Calcarine fissure	18 weeks to 19 weeks 6 days (dot pattern)
Cingulate sulcus	20 weeks to 21 weeks 6 days (dot pattern)
Sylvian fissure	20 weeks to 21 weeks 6 days (obtuse angle)
Convexity sulci	25 weeks and beyond

## Discussion

CNS malformations contribute significantly to congenital abnormalities. Among them are neural tube defects, which contribute to around two cases per 1,000 births [13]. Fetal CNS anomalies have been detected by ultrasound in the last 30 years, which has continued to be the key tool in the diagnosis of CNS anomalies. An elaborated evaluation of fetal CNS in neurosonography requires expertise and advanced ultrasound machines. In recent years, fetal ultrasonography is accompanied by 3D ultrasonography, which demonstrates better visualization of structures [13].

The functional integrity and maturity of the fetal brain has been assessed based on myelination and sulcal development as it could be well correlated with psychomotor development [14,15]. However, in the fetus, myelination occurs incompletely starting from the thalamus and brain stem. Hence, sulcal development is suggested to be a more precise indicator of fetal brain maturation in utero compared to myelination. As the gestational age advances, the sequential development of sulcation also progresses. Hence, we attempted the study of serial appearance of various fetal cerebral sulci on ultrasonography.

The present study included 241 antenatal mothers. Majority (42.32%, n=102) of them were between 23 and 27 years of age, and 34.85% (n=84) were between 18 and 22 years of age. Among 241 fetuses, 35 (14.52%) were studied between 18 weeks and 19 weeks 6 days, 20 weeks and 21 weeks 6 days, 22 weeks and 23 weeks 6 days, 26 weeks and 27 weeks 6 days, 28 weeks and 29 weeks 6 days, and 30 weeks and 32 weeks gestation each, and 31 fetuses (12.86%) were studied between 24 weeks and 25 weeks 6 days. The mean period of gestation as per dating scan was 24.99 ± 4.21 weeks, ranging from 17.57 to 33.71 weeks, and as per the present ultrasound it was 24.99 ± 4.13 weeks, ranging from 18 weeks to 32 weeks.

The cerebral sulci that were studied in our study were POF, CF, CS, insula/ Sylvian fissure, and convexity sulci. POF was the first fissure to develop and was always present at 18 weeks to 19 weeks 6 days as a V-shaped fissure and later developed into a Y-shaped configuration by 21-22 weeks. CF was the next fissure to develop and was seen by 18 weeks to 19 weeks 6 days as a dot pattern, and then developed to form a V-shaped indentation by 20 weeks to 21 weeks 6 days and finally developed into a Y-shaped configuration by 22 weeks to 23 weeks 6 days. CS was seen later in the gestation by 21 weeks as a dot, and then developed into a V-shaped indentation by 24 weeks to 25 weeks 6 days and finally as a Y-shaped configuration by 28 weeks to 29 weeks 6 days. SF initially appeared as a smooth surface, and then operculized to form an obtuse angle with the adjacent temporal lobe by 20 weeks to 21 weeks 6 days and further operculized to form an acute angle by 24 weeks to 25 weeks 6 days. Convexity sulci were visualized later in the gestation, beyond 26 weeks to 27 weeks 6 days.

In a serial ultrasound study by Cohen-Sacher et al. [4], antenatal ultrasonography of 22 pregnant women was performed every 2 weeks from 18 weeks of gestation until term and concluded that the first sulci can be seen as early as 18 weeks of gestation. Other major sulci such as cingulate and calcarine sulci were seen between 22 and 24 weeks of gestation. By 30-32 weeks, most of the main sulci were seen. These findings were in conjunction with the findings noted in our study.

An anatomical study conducted by Nishikuni and Ribas [16] studied 107 human brain specimens and reported that POF was present in 50% of fetuses at 17 weeks of gestation, CF was present in 63% of fetuses at 17 weeks, CS was present in 80% fetuses at 19 weeks, and SF was present in 100% fetuses at 29 weeks ± 2 weeks.

Limitations and recommendations

The present study was based on ultrasound, which is observer-dependent, and therefore further correlation with 3D sonography and MRI is recommended. Shadowing from the fetal skull can hinder visualization of the sulci in the hemisphere nearer to the transducer in later gestational age. Post-natal follow-up would increase the efficacy of the study.

## Conclusions

Neuronal migration disorders result in a wide spectrum of sulcal and gyral malformations whose clinical manifestations are deleterious and include severe psychomotor retardation, cognitive and developmental delays, motor deficits, seizures, and failure to thrive. Therefore, it is crucial to diagnose these conditions early in pregnancy for accurate antenatal counselling and optimization of obstetric management.

Ultrasonography, which is the commonest modality used in assessing the development and growth of the fetus, can also be used to identify, familiarize, and provide a standard reference to assess normality of fetal sulcations. In our study, we observed that POF was the first fissure to develop and was always present at 18 weeks of gestation as a V-shaped indentation. CF was the next fissure to develop and was seen by 18 weeks as a dot. CS appeared later by 21 weeks. SF was initially present as a smooth surface and then operculized to form an obtuse angle by 20 weeks and then an acute angle by 24 weeks. Convexity sulci were confidently visualized later in the gestation, beyond 26 weeks. This study can not only help in familiarizing the appearance patterns of major cerebral sulci as per their gestational age but also has a vital role in early suspicion and detection of neuronal migration disorders, when present. Further research using 3D ultrasonography and fetal MRI techniques will increase the sensitivity of our study and further strengthen our results. Post-natal follow-up of the affected fetuses will also help in confirming the findings on antenatal studies.

## References

[1] Usha M, Sudharani K, Ratnachary P (2015). Development of sulci and gyri at different foetal age groups. J Evid Based Med Healthc.

[2] Afif A, Trouillas J, Mertens P (2015). Development of the sensorimotor cortex in the human fetus: a morphological description. Surg Radiol Anat.

[3] Chen X, Li SL, Luo GY (2017). Ultrasonographic characteristics of cortical sulcus development in the human fetus between 18 and 41 weeks of gestation. Chin Med J (Engl).

[4] Cohen-Sacher B, Lerman-Sagie T, Lev D, Malinger G (2006). Sonographic developmental milestones of the fetal cerebral cortex: a longitudinal study. Ultrasound Obstet Gynecol.

[5] Alves CM, Araujo Júnior E, Nardozza LM (2013). Reference ranges for fetal brain fissure development on 3-dimensional sonography in the multiplanar mode. J Ultrasound Med.

[6] Rolo LC, Araujo Júnior E, Nardozza LM, de Oliveira PS, Ajzen SA, Moron AF (2011). Development of fetal brain sulci and gyri: assessment through two and three-dimensional ultrasound and magnetic resonance imaging. Arch Gynecol Obstet.

[7] Pistorius LR, Stoutenbeek P, Groenendaal F, de Vries L, Manten G, Mulder E, Visser G (2010). Grade and symmetry of normal fetal cortical development: a longitudinal two- and three-dimensional ultrasound study. Ultrasound Obstet Gynecol.

[8] Pingping X, Dirong Z, Yu S (2021). Evaluation of the depth and width of normal fetal sylvian fissure by trans-cerebellar section [PREPRINT]. MedRxiv.

[9] Toi A, Lister WS, Fong KW (2004). How early are fetal cerebral sulci visible at prenatal ultrasound and what is the normal pattern of early fetal sulcal development?. Ultrasound Obstet Gynecol.

[10] Quarello E, Stirnemann J, Ville Y, Guibaud L (2008). Assessment of fetal Sylvian fissure operculization between 22 and 32 weeks: a subjective approach. Ultrasound Obstet Gynecol.

[11] Alonso I, Borenstein M, Grant G, Narbona I, Azumendi G (2010). Depth of brain fissures in normal fetuses by prenatal ultrasound between 19 and 30 weeks of gestation. Ultrasound Obstet Gynecol.

[12] Gnanasigamani S, Alagappan P, Chellathurai A (2018). Assessment of normal cerebral sulcal development in foetus using MRI. IOSR J Dent Med Sci.

[13] International Society of Ultrasound in Obstetrics & Gynecology Education Committee (2007). Sonographic examination of the fetal central nervous system: guidelines for performing the 'basic examination' and the 'fetal neurosonogram'. Ultrasound Obstet Gynecol.

[14] Barkovich AJ, Kjos BO, Jackson DE Jr, Norman D (1988). Normal maturation of the neonatal and infant brain: MR imaging at 1.5 T. Radiology.

[15] Fleischer AC. (2002). Ultrasonography of the prenatal and neonatal Brain. J Perinatol.

[16] Nishikuni K, Ribas GC (2013). Study of fetal and postnatal morphological development of the brain sulci. J Neurosurg Pediatr.

